# Premature Baldness

**Published:** 1905-09-16

**Authors:** 


### PREMATURE BALDNESS.

An anonymous correspondent, writing to the *Lancet*,[1] advises the following procedure in cases of premature baldness: The hair to be kept short and the head to be washed every morning with a pint of warm water containing one drachm of izal and applied without soap and by means of a soft sponge; after the hair has dried there must be well rubbed into the scalp a pomade consisting of boric acid ointment, salicylic acid ointment, and lanoline. Then the scalp is rubbed with a soft towel until all greasiness is removed.

[1] Aug. 26, 1905.

